# Floristic Diversity and Indicator Species Analysis Along Altitudinal Gradients of the Upper Indus Basin, Northern Pakistan

**DOI:** 10.1002/ece3.73228

**Published:** 2026-03-08

**Authors:** Adam Khan, Sidra Saleem, Sahar Zaidi, Zeeshan Ahmad, Hamada E. Ali

**Affiliations:** ^1^ Department of Botany University of Lakki Marwat Lakki Marwat Khyber Pakhtunkhwa Pakistan; ^2^ Department of Botany Abdul Wali Khan University Mardan Mardan Khyber Pakhtunkhwa Pakistan; ^3^ Department of Botany Federal Urdu University of Arts, Science and Technology Karachi Pakistan; ^4^ Laboratory of Tropical Forest Ecology Xishuangbanna Tropical Botanical Garden, Chinese Academy of Sciences Menglun Mengla, Yunnan China; ^5^ Department of Biology College of Science, Sultan Qaboos University Muscat Oman; ^6^ Botany and Microbiology Department, Faculty of Science Suez Canal University Ismailia Egypt

**Keywords:** CCA and DCA ordinations, cluster analysis, indicator species analysis, northern Pakistan, upper Indus Basin

## Abstract

Understanding how altitudinal gradients influence floristic diversity and indicator species is essential for unlocking the ecological dynamics of biodiversity‐rich regions. We examined the floristic diversity, communities' formation and their respective indicator species across defined altitudinal zones of the Upper Indus Basin region in Indus Kohistan Valley, northern Pakistan. Vegetation was sampled along transects ranging from 1957 to 3380 m using quadrat, with a total of 600 quadrats from 30 different sites surveyed during the summer season (June–August). Most plants belonged to family Asteraceae, Pinaceae, Lamiaceae and Berberidaceae, with chamaephytes as the dominant life forms, followed by geophyte and phanerophytes. Cluster Analysis classified the vegetation into three communities: *Taxus*‐*Rumex*‐*Mentha* (TRM)*, Pinus*‐*Indigofera*‐*Leontice* (PIL)*,* and *Pinus*‐*Phyllanthus*‐*Valeriana* (PPV). Species attributes plots identified based on Canonical Correspondence Analysis demonstrated that TRM community is primarily influenced by calcium, pH and salinity. The PIL community is limited by potassium, oxygen reduction potential, sand and silt while the PPV community by sodium, MWHC, soil moisture and carbon content. Tukey showed that the TRM community had the highest mean dominance, the PIL community exhibited the highest Simpson, Shannon and Evenness indices, and the PPV community had the lowest values, indicating that soil properties and microclimatic factors along the altitudinal gradients shape the species composition and association. Detrended correspondence analysis explained a total of 23.89% of the variance, as the first axis illustrated the maximum gradient length (3.07) further strengthening the influence of environmental variables on species distribution and association. The DCA indicated that environmental variables such as salinity, pH, carbon content, soil texture, and calcium substantially influenced species distribution and association, a pattern supported by the Mantel test. It is recommended that reforestation efforts should prioritize the PPV community at high‐altitude zone (2390–3380 m) and consider sodium, MWHC, soil moisture and carbon content when selecting suitable indicator species for restoration.

## Graphical abstract

Altitudinal gradients substantially influence floristic diversity and indicator species. Three vegetation communities were identified, each shaped by specific environmental variables. Mantel test shows that altitude, Ca, K, Na are key drivers of species diversity and association.

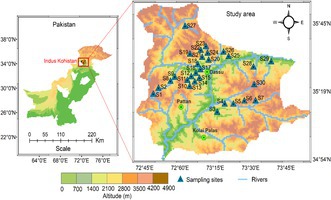

## 1 Introduction

The upper Indus Basin located within the Kohistan Valley of Hindu Kush‐Karakoram‐Himalayan (HKH) mountainous regions supports unique altitudinal gradients and diverse vegetation. The study of floristic diversity along the altitudinal gradients is fundamental for understanding the ecological indicator species and ensuring sustainable ecosystem management. Environmental gradients have a substantial influence on species composition and distribution over a relatively short spatial scale (Aljasmi et al. 2021; Anwar et al. 2023). Vegetation structure is mostly dependent on these environmental gradients, which support diverse ecosystems throughout various regions (Fischer and Mölder 2017; Rahman et al. 2022). While numerous studies have analyzed vegetation‐environmental relationships and indicator species analysis (ISA) in alpine, boreal, and temperate regions (Shaheen et al. 2011; Marchetti et al. 2018; Gillani et al. 2024). These frameworks have yet to be applied to the vegetation of the upper Indus Basin in the Indus Kohistan Valley, northern Pakistan. In this region, the interaction of HKH topography, marked altitudinal variation, and soil properties generates unique ecological gradients. However, indicator species analysis and the relationship of floristic diversity with soil variables remain limited.

The soil variables provide synoptic information about vegetation dynamics and its association (Khan et al. 2020; Onditi et al. 2024). Qualitative attributes of vegetation dynamics are significantly influenced by soil, topography, altitude, and to some extent biotic factors (Ilyas et al. 2012; Haq et al. 2024). Plant species exhibit diverse climatic conditions, but their distribution pattern is mainly modulated by natural habitats and soil nutrients, highlighting their ecological vigor (Zeb et al. 2017, 2020). Both physical and chemical characteristics have a significant influence on species abundance (Yang et al. 2018). The altitudinal gradients provide valuable insight to examine the variation of abiotic factors and their influences on plant species associations (Upadhyay et al. 2018; Rahman et al. 2020). Studies evidence that topographic variables such as altitude and slope play a substantial role in species assemblage and distribution (Haq and Badshah 2021). Altitudinal variation results in dramatic changes in climate and discrete zones within mountainous ecosystems, each harboring unique species associations and distributions (Pauli and Halloy 2019). In addition, distinct environmental factors and soil nutrients across the altitudinal gradients also support distinct plant communities and their associated indicator species (Ahmad et al. 2016; Abbas et al. 2020). The indicator species reflect a distinct set of environmental conditions (Burgass et al. 2017; Wu et al. 2020). In addition, the understanding of plant species association and their relationship with biotic and abiotic factors within an ecosystem has been recognized as essential in modern ecological research (Zhao et al. 2018).

Multivariate analysis provides a powerful framework to evaluate the complex vegetation dataset and explore spatial and temporal pattern of biodiversity along environmental gradients (Dray et al. 2012; Iqbal et al. 2022). In mountainous ecosystems, such as the upper Indus Basin in Indus Kohistan Valley, vegetation patterns are largely controlled by these environmental gradients (Khan et al. 2020; Iqbal et al. 2022; Anwar et al. 2023). Multivariate approaches such as classification and ordination of vegetation allow the ecologist to determine the vegetation‐environment relationship and indicator species analysis for specific habitats (Dufrêne and Legendre 1997; Hussain et al. 2019; Anwar et al. 2023). The life form also acts as a natural proxy for evaluating micro‐ and macroclimate which offers additional insight of vegetation‐environment relationship (Shimwell 1971; Saxena et al. 1982).

The upper Indus Basin in Indus Kohistan Valley, northern Pakistan is characterized by diverse climatic zones and rugged topography. The majestic HKH in this region supports rich biodiversity with both ecological and medicinal values. In addition, life form, floristic diversity, and ecological indicator species are directly associated with altitudinal gradients. In this context, we hypothesized that the turnover in floristic composition across the altitudinal gradient of the Indus Kohistan Valley is primarily limited by shifts in soil variables. These variations in soil variables lead to the appearance of distinct indicator species within each community. Therefore, this study focuses on floristic diversity and ISA along the altitudinal gradients of upper Indus Basin in Indus Kohistan Valley, northern Pakistan. Specifically, this study aims to: (1) assess the floristic diversity, life form and habitat of plant species along different altitudinal gradients; (2) quantify the influence of soil variables on species association and distribution; and (3) identify indicator species within community and their relationship with soil variables.

## 2 Materials and Methods

### 2.1 Study Area

**FIGURE 1:**
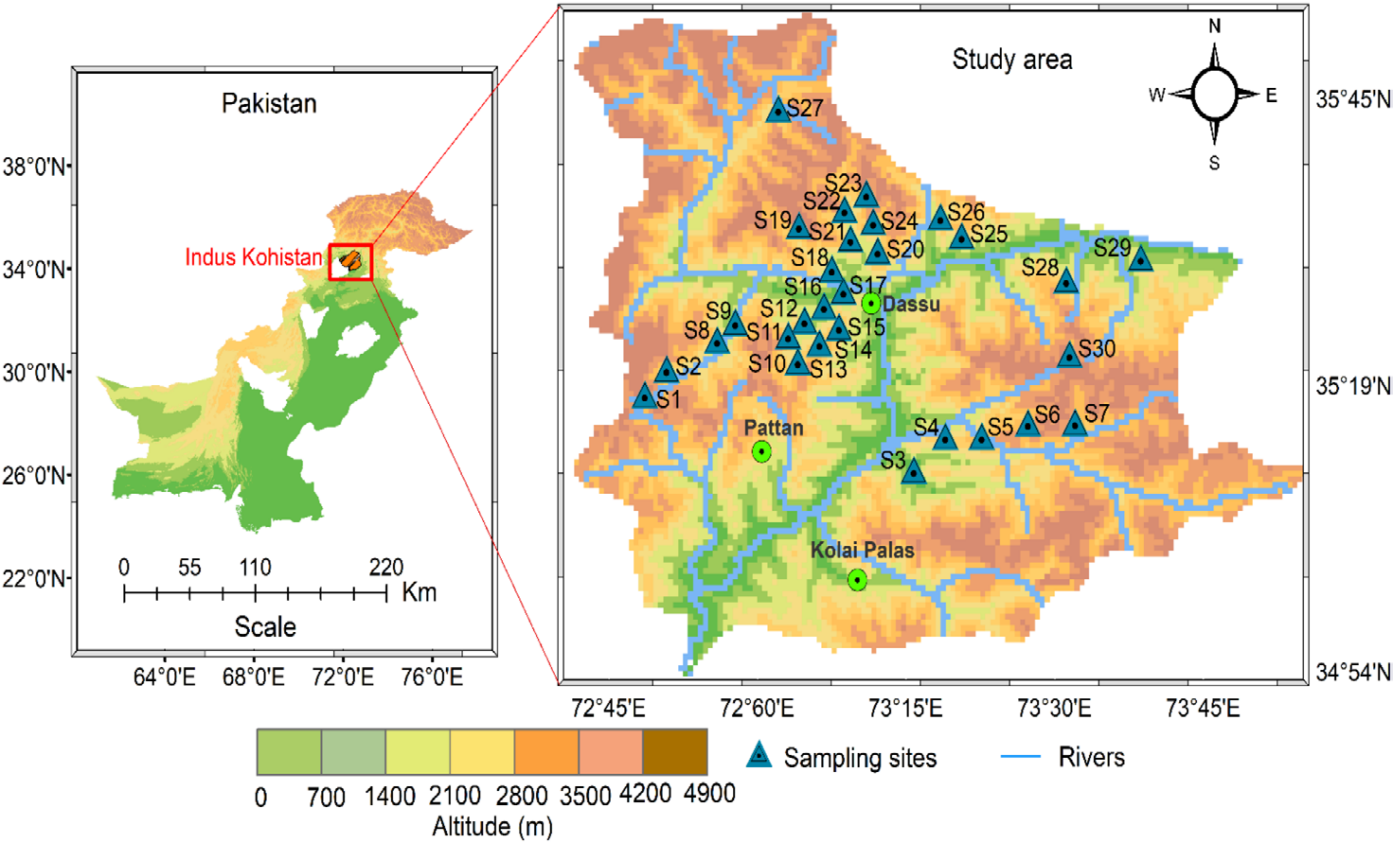
Map of the study area generated by Arc GIS, showing the sampling sites of vegetation across the Indus River and its territory, northern Pakistan.

The study area is located in northern Pakistan (34.54°–35.52° N, 72.43°–73 57° E) covering an area of 2893 km^2^ (Figure 1). The region spreads over the Shangla district to Chilas, covering upper and lower Indus Kohistan districts and Kolai‐Palas district. Geographically, the region is bordered to the north by Gilgit‐Baltistan, to the east by Mansehra district, and to the west by Shangla and Battagram districts. The HKH ranges in the region play a substantial role in regulating the microclimate conditions via shaping distinct associations and ecological indicator species. However, till now, little literature is available in terms of plant biodiversity (Khan, Ahmed, et al. 2016; Khan et al. 2020). This highlights the importance of ecological studies to understand the relationship between environmental factors and vegetation and indicator species. The bank of the Indus River harbors conifer forests dominated by *Cedrus deodara* , *Pinus wallichiana* , *Abies pindrow*, *Picea smithiana* , and *Pinus gerardiana* (Khan, Ahmed, et al. 2016). In addition, *Taxus wallichiana*, *Juniper excelsa*, and *Quercus baloot* are also spread over the valley (Islam et al. 2021; Amin et al. 2024).

### 2.2 Sampling Procedure and Laboratory Analysis

Vegetation sampling was conducted using quadrats along altitudinal transects in the upper Indus Basin of the Indus Kohistan Valley, northern Pakistan, covering an altitude range of 1957–3380 m. To ensure local variation in vegetation, transects were taken along the slope of the mountains of each sampling site. Quadrat sizes were selected according to vegetation type, with larger quadrats (10 × 10 m^2^) used for trees, medium (5 × 5 m^2^) for shrubs and small (1 × 1 m^2^) for herbaceous vegetation. In order to ensure maximum plant visibility, the sampling was performed during the summer season (June–August). At each transect, a total of 20 quadrats were established each for trees, shrubs and herbaceous vegetation. The distance between sampling sites was spatially separated by a minimum distance of 15 km. Voucher plant specimens of wild plants were collected, tagged, air‐dried and pressed following standard procedures (Ali 2008; Khan et al. 2020). Since most plant specimens are generally identified on their reproductive structures, in the field we ensured to collect those parts of plants which bear flowers or seeds. The collected plant specimens were identified by using the *Flora of Pakistan* (Ali 1978). The identified plant specimens were deposited in the herbarium of the department of Botany, Government college University Lahore, as a reference for future research. The voucher number for each collected specimen is provided in Table S1. Vegetation densities (plants ha^−1^) of the sampled sites were determined for each life form, and the co‐occurrence of species was analyzed within quadrats and across transects to examine how edaphic factors impact the species distribution and the occurrence of indicator species.

Across all sampling sites, three soil samples were taken from lower, middle, and upper elevations using a soil auger and mixed into a composite sample for various environmental factor analysis. The composite soil samples were sieved through a 2 mm sieve, and a physiochemical analysis of these soil samples was performed following standard protocols. For instance, total organic matter (TOM) was calculated utilizing the loss on ignition method by heating the soil samples in a furnace at 550°C (Heiri et al. 2001). For the determination of maximum water holding capacity (MWHC), 5‐g composite air‐dried soil samples were placed in tins with perforated bottoms and saturated with water. The soil was subsequently oven‐dried, and the % MWHC was assessed. For the determination of salinity, pH, total dissolve solid (TDS), and electrical conductivity (EC), a soil–water suspension (1:5, w/v) was prepared by mixing the obtained soil with distilled water and gently shaken to confirm the uniformity. The suspension was allowed to equilibrate, after which readings were noted utilizing a calibrated Hanna multi‐parameter probe (Model HI9828). One gram of air‐dried soil was digested with 10 mL of concentrated H_2_SO_4_ in a Kjeldahl digestion flask until a clear solution formed. To convert the nitrogen into ammonium sulfate, the cooled solution was diluted in distilled water. The ammonium was condensed into a boric acid solution and titrated with HCl. The total nitrogen was measured from the acid volume consumed (Khan et al. 2020). The soil essential elements and heavy metals such as sodium, magnesium, calcium, zinc, and nickel were estimated with an Atomic Absorption Spectrophotometer (model PG‐990) (Gungshik et al. 2021).

### 2.3 Data Analysis

The species–area curve was used to evaluate representation of species in each community along the altitudinal gradient (Anwar et al. 2023). Using Cluster Analysis (CA), the vegetation of the sampling sites was classified into different plant communities using presence‐absence (1, 0) data and Sorensen measures distance measurement through PC‐ORD version 5 (McCune 1986; Mehmood et al. 2021). Similarly, the Two‐Way Cluster Analysis (TWCA) represented the presence and absence of species in each community (Ahmad et al. 2016). The Indicator Species for each community were determined through indicator species analysis (ISA) (Lepš and Šmilauer 2003) via utilizing a Monte Carlo test with a threshold limit of 20% significance (*p* ≤ 0.05) following (Dufrêne and Legendre 1997). The vegetation‐environmental variables relationship was assessed by using Canonical Correspondence Analysis (CCA) and Detrended Correspondence Analysis (DCA) through CANOCO version 5 software. This analysis was conducted via utilizing species density and measured environmental data (Khan et al. 2020; Anwar et al. 2023; Tu et al. 2024). To minimize multicollinearity and retain ecologically meaningful, non‐redundant ecological variables, highly correlated environmental variables (threshold limit, *r* = 0.8) were eliminated prior to analysis. Before performing the analysis, the species data were arranged per requirement of the CANOCO version 5 (Lepš and Šmilauer 2003; Iqbal et al. 2022).

### 2.4 Diversity Pattern and Their Connection With Environmental Variables

The diversity indices of all sampled sites were evaluated through PAST software (version 4.12), employing dominance, Simpson, Shannon, and Evenness to determine species diversity along the Indus river (Hammer and Harper 2001). The measured Dominance and Simpson indices show the characterization of dominance. The Shannon index demonstrates a combination of species evenness and richness, whereas the Evenness index reflects the homogeneity of the plant community, offering a comprehensive insight into species distribution patterns. In order to provide the variation in diversity across the sampling site we represented the obtained diversity indices through Ridgeline plots via utilizing OriginPro software.

## 3 Results

A total of 78 plant species from 40 different families were recorded from the studied Upper Indus Basin, Hindu Kush‐Himalaya region. The majority of the recorded species belong to family Asteraceae (11 species, 14.1%), followed by Pinaceae (7 species, 9.1%), Lamiaceae (6 species, 8.1%), and Berberidaceae (5 species, 6.4%) (Table S1). Based on habitat, most of the species were herbaceous (59 species, 75.6%), followed by shrubs (12 species, 15.4%), and trees (7 species, 8.9%). The dominant life form was chamaephytes (25 species, 32.1%), followed by geophytes (17 species, 21.8%), and phanerophytes (16 species, 20.5%).

### 3.1 Floristic Composition and Key Indicator Species

**FIGURE 2:**
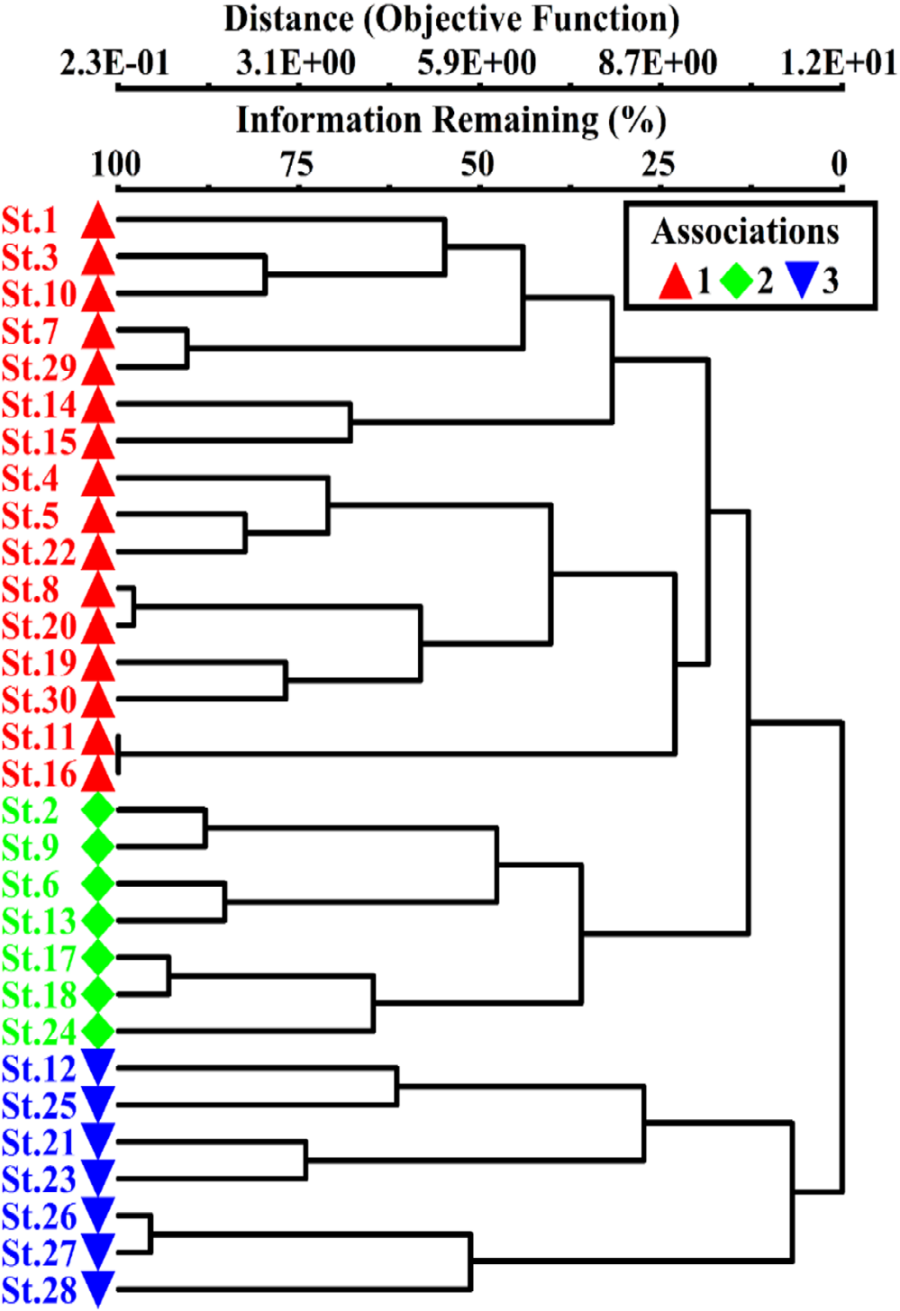
Cluster analysis (CA) dendrogram classified the sampling sites into three major plant communities along the Indus River, northern Pakistan.

The species area curves were constructed to evaluate sample size adequacy and to understand the associated environmental variables. The results reveal a substantial change in number of species from sampling sites 3 to 29. This change highlights the connection of species distribution with altitudinal gradient (Figure S1). The Ward's cluster analysis (CA) classified vegetation into three distinct communities such as *Taxus‐Rumex‐Mentha* (TRM), *Pinus‐Indigofera‐Leontice* (PIL), and *Pinus‐Phyllanthus‐Valeriana* (PPV) communities (Figure 2). The species distribution in the identified communities was illustrated by Two‐Way Cluster Analysis (TWCA) dendrogram (Figure S2). Detail of each community is as follows:

#### 3.1.1 TRM Community

**FIGURE 3:**
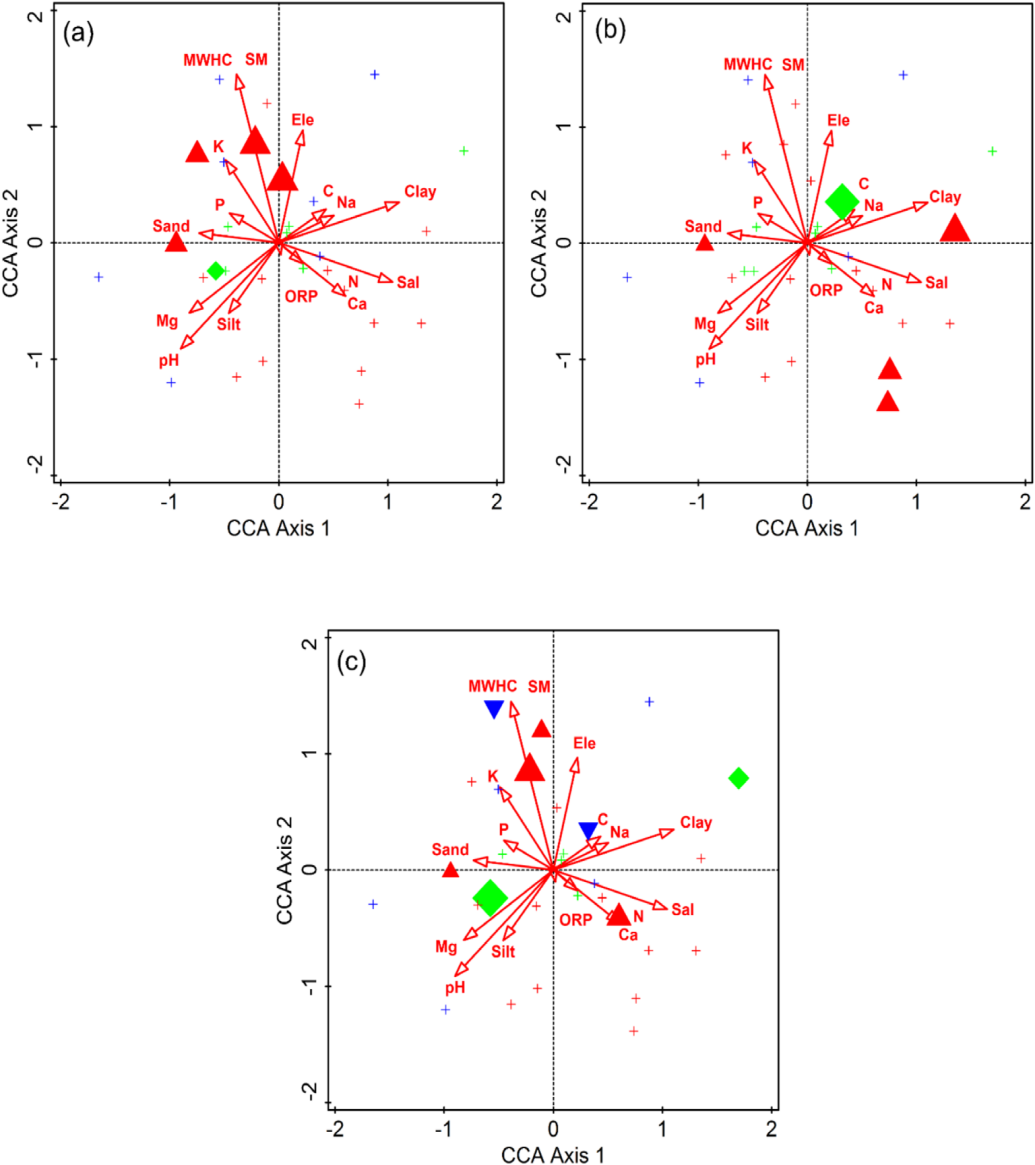
Attribute plots for indicator species (a) *Taxus* wallichiana, (b) *Rumex nepalensis*, and (c) *Mentha Pulegium* in relation to different environmental variables along the Indus River, northern Pakistan.

This community was recorded at 16 different sampling sites within the study area, spanning an altitude range of 1957–3044 m. Based on ISA, the key indicator species of this community were *Taxus wallichiana, Rumex nepalensis*, and *Mentha Pulegium* (Figure 3). These species were the indicators of higher concentration of calcium (0.7–28 mg g^−1^), pH (6.8–7.8) and salinity (0.1%–0.2%) (Table 1). The dominant tree species in this communities was *Cedrus deoara* (294.7 plant ha^−1^). The most abundant shrub species were *Berberis brandisiana* (91.1 plant ha^−1^) and *B. lyceum* (89.7 plant ha^−1^), whereas top herbaceous species were *Conyza bonarienss* (144.9 plant ha^−1^) and *Thymus linearis* (99.2 plant ha^−1^). Rare species of this community included *Geranium wallichianum* (1.7 plant ha^−1^), *Hypericum perforatum* (1.6 plant ha^−1^), *Verbascum thapsus* (1.5 plant ha^−1^), *Corydalis govaniana* (1.4 plant ha^−1^), *Heteropappus altaicus* (1.2 plant ha^−1^). This community was influenced by numerous edaphic factors, which exhibited various ranges of ORP (48.8–113.8), MWHC (2.5%–14.4%), soil moisture (5%–28.8%), carbon content (0.5%–2.9%), sand (30%–62.8%), silt (29.4%–43.6%), clay (0.4%–34.8%), magnesium (0.6–2.7 mg g^−1^), sodium (0.2–21.7 mg g^−1^), potassium (0–13.1 mg g^−1^), nitrogen (2.4%–3.3%), and phosphorus (3–11 mg g^−1^).

**TABLE 1:** Result of indicator species analysis (ISA), highlighting the most indicator species (bolded) for each plant community at threshold limit of 20% indicator value (IV) and Mont Carlo test of significance for observed maximum indicator value (*p* ≤ 0.05).

S. No	Code	*Taxus‐Rumex‐Mentha* Community demonstrate by Ca			Pinus‐Indigofera‐Leontice Community denoted by K			Pinus‐Phyllanthus‐Valeriana Community denoted by Na		
		Max grp	IV	p*	Max grp	IV	p*	Max grp	IV	p*
1	*Angelica archangelica*	13	16.1	0.8628	2	17.4	0.775	5	28.1	0.4347
2	*Abies pindrow*	2	28.5	0.5107	3	29.7	0.3749	5	18.1	0.8254
3	*Adiantum venustum*	13	58.3	0.0751	3	34.2	0.4879	1	20.9	0.6403
4	*Anaphalis nepalensis*	13	20.1	0.6641	2	16.6	0.8516	11	42.7	0.1096
5	*Aquilegia nivalis*	13	41.5	0.1532	0	26.3	0.5609	5	55.6	0.0652
6	Arenaria serpyllifolia	2	15.9	0.9128	21	26.3	0.4557	1	19.9	0.7065
7	*Artemisia sieversiana*	13	29.1	0.3239	0	22	0.5445	2	27.9	0.3619
8	*Asparagus filicinus*	4	18.7	0.7025	13	44.5	0.1238	1	27.9	0.3697
9	*Astragalus himalayannus*	8	22.5	0.6523	2	42.9	0.1092	2	23.4	0.1216
10	*Berberis brandisiana*	4	26.2	0.5439	2	35.9	0.3381	1	14.6	0.1834
11	*Berberis lycium*	8	21	0.7057	13	16.2	0.9246	2	20.6	0.6343
12	*Berberis vulgaris*	1	17.1	0.8292	13	32.9	0.2773	1	15.3	0.9268
13	*Bergenia cilliata*	4	23.9	0.5609	15	20.7	0.6227	1	21.7	0.6791
14	*Bistorta amplexicaulis*	13	22.1	0.6721	0	22.8	0.6397	8	22.3	0.5469
15	*Cedrus deodara*	2	21.3	0.6707	13	23.8	0.2422	1	23.6	0.3039
16	*Cirsium arvense*	13	30.5	0.4971	13	37.1	0.2967	5	66.7	0.0442
17	*Clematis barbellata*	13	40.2	0.1098	21	16	0.9306	11	17.3	0.6497
18	*Clematis tangutica*	1	33.3	0.3201	13	23	0.885	11	43	0.176
19	*Cleome brachycarpa*	0	14.7	0.9354	21	15.6	0.9268	8	47.2	0.0888
20	*Cleome scaposa*	8	11	0.9056	12	29.9	0.4957	8	59.6	0.0636
21	*Conyza bonarienss*	2	39.4	0.191	21	25.5	0.6015	8	29.9	0.4101
22	*Corydalis govaniana*	13	47.5	0.1892	12	33.3	0.7033	2	14	0.9154
23	*Cousinia thomsonii*	4	25.9	0.4857	4	56.5	0.068	5	16.9	0.7361
24	*Cynoglossum lanceolatum*	0	23.6	0.5461	13	25.3	0.4647	9	19.3	0.5595
25	*Desmodium gangeticum*	4	22.3	0.5293	2	20	0.6469	1	50.5	0.0608
26	*Fragaria nubicola*	4	22.2	0.6107	0	26.9	0.3399	1	59.5	0.0492
27	*Fragaria vesca*	1	16.7	0.5821	13	50	0.3381	5	33.3	0.5443
28	*Geranium himalayense*	1	29.8	0.3997	13	33.3	0.4373	5	29.6	0.5401
29	*Geranium wallichianum*	2	53	0.0858	13	15.9	0.8724	5	11.5	0.9562
30	*Hedera nepalensis*	4	62	0.0334	3	26	0.3139	5	30.8	0.2272
31	*Heliotropium strigosum*	2	15	0.9302	4	28.9	0.3009	7	12.8	0.9146
32	*Heteropappus altaicus*	1	13.8	0.7902	2	9.9	0.963	1	28.1	0.5871
33	*Hyosymus nigir*	8	25.3	0.3961	0	18.6	0.6481	2	47.4	0.087
34	*Hypericum perforatum*	1	16.7	0.5801	15	48.2	0.1542	11	50	0.1553
35	*Impatiens bicolor*	8	16.2	0.7415	2	12.6	0.9332	8	23.6	0.3977
36	*Impatiens edgeworthii*	0	19.7	0.6523	2	9.7	0.9882	11	17.9	0.6433
37	*Indigofera heterantha*	4	18.4	0.7682	2	16.2	0.9322	1	25	0.4323
38	***Indigofera trita*** ^***2***^	2	21.8	0.5357	**13**	**46.9**	**0.0446**	8	16.3	0.8344
39	***Leontice eversmamnii*** ^***2***^	0	15.2	0.9064	**15**	**75.5**	**0.0062**	8	19.5	0.6457
40	*Leucas nutans*	0	29.6	0.4397	21	16.8	0.8544	1	26.2	0.4607
41	*Lolium multiforum*	4	16.8	0.7816	21	14.4	0.8588	7	17.6	0.6675
42	*Mentha longifolia*	0	8.3	1	21	33.3	0.6963	7	20	0.7538
43	***Mentha Pulegium*** ^***1***^	**13**	**78.3**	**0.0216**	12	45.3	0.1488	5	26.5	0.5341
44	*Mentha royleana*	4	16.3	0.8772	12	27.1	0.3421	8	37.4	0.2172
45	*Micromeria biflora*	1	20.8	0.7504	2	40.5	0.1426	1	43.8	0.1312
46	*Myosotis alpestris*	3	40.2	0.1716	2	14.3	1	1	33.3	0.5417
47	*Myosotis sylvatica*	13	18.3	0.7931	4	28.7	0.3315	9	33.7	0.1398
48	*Olea glandulifera*	1	12.7	0.9576	21	16.8	0.8198	7	7.1	0.9994
49	*Orthosiphon palliidus*	8	17.6	0.8432	13	22.7	0.5941	9	34.5	0.1546
50	*Parasenecio delphiniifolius*	4	12	0.9058	3	33.3	0.7121	5	33.3	0.5395
51	***Phyllanthus parvifolius*** ^***3***^	2	21.5	0.6553	13	28.5	0.224	**11**	**39.4**	**0.0123**
52	*Picea smithiana*	13	13.6	0.9544	12	31.9	0.3331	5	36.3	0.2765
53	***Pinus gerardiana*** ^***3***^	0	26.1	0.4865	2	9	0.7153	**8**	**68.4**	**0.0374**
54	***Pinus wallichiana*** ^***2***^	1	20.6	0.6697	**13**	**32**	**0.0521**	5	29.4	0.2042
55	*Plantago amplexicaulis*	8	30.6	0.3639	15	31.9	0.4793	7	14.6	0.8624
56	*Poa polycolea*	13	17.8	0.7119	2	32.9	0.2186	11	21.7	0.4785
57	*Podophyllum emodi*	2	28.3	0.3875	0	53.3	0.0318	2	41.6	0.1088
58	*Pteridium aquilinum*	13	57.9	0.0678	12	34.8	0.2386	11	28.8	0.3229
59	*Quercus baloot*	4	27.1	0.4829	3	10	1	1	18.9	0.6337
60	*Ranunculus laetus*	2	24.5	0.5531	0	22.3	0.7231	2	19	0.7413
61	*Rheum spiciforme*	4	24.5	0.5819	2	17.6	0.9238	1	26.8	0.4215
62	*Rhynchosia minima*	13	15.1	0.8996	0	12.2	0.949	2	19.4	0.6017
63	*Rosa webbiana*	4	28.1	0.4301	3	23.2	0.6551	7	24.7	0.5273
64	*Rumex hastatus*	1	11.2	0.9916	2	16	0.9558	9	23.2	0.4097
65	***Rumex nepalensis*** ^***1***^	**8**	**70.8**	**0.0034**	4	50.6	0.0754	8	16	0.8504
66	*Satyrium nepalense*	1	14.6	0.8868	2	18.7	0.8252	11	37.1	0.1588
67	*Saussurea lappa*	2	54.5	0.0796	0	21.8	0.5505	7	18.6	0.6229
68	*Senecio deodrans*	13	28.5	0.3565	15	81.1	0.0134	2	34.1	0.1908
69	*Sida cordifolia*	3	24.1	0.5875	0	43.2	0.1562	7	29.7	0.3475
70	*Silene vulgaris*	13	44.2	0.1158	12	47.9	0.1286	5	20.4	0.7073
71	Sisymbrium irio	8	20.6	0.7143	15	24.9	0.4983	11	23.9	0.4193
72	*Solidago virgaurea*	1	16.7	0.5961	12	27.1	0.4621	9	50	0.1646
73	***Taxus wallichiana*** ^***1***^	**13**	**44.8**	**0.0098+**	12	18	0.8564	5	31.6	0.3213
74	*Thymus linearis*	13	32.1	0.3223	21	21.9	0.7934	5	42.3	0.125
75	***Valeriana jatamansi*** ^***3***^	1	8.4	0.995	4	15.6	0.7836	**11**	**98.7**	**0.0032**
76	*Verbascum Thapsus*	8	51.8	0.057	3	33.4	0.2703	9	17	0.7051
77	*Viola betonicifolia*	2	18.7	0.7031	0	35.6	0.2579	2	15.1	0.8816
78	*Withania coagulans*	3	29.4	0.4217	13	32	0.052	2	28.6	0.3567

#### 3.1.2 PIL Community

**FIGURE 4:**
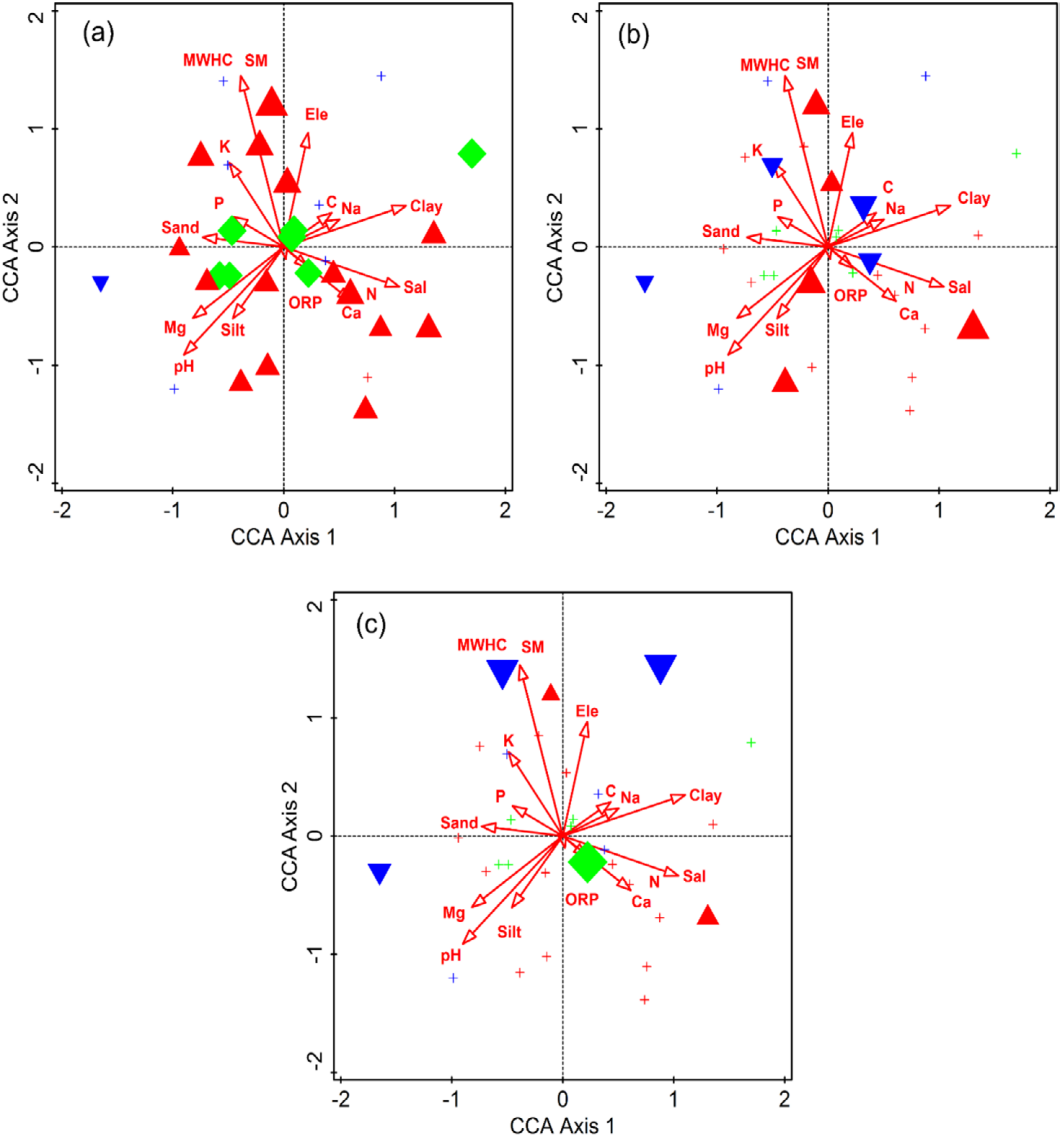
Attribute plots of indicator species (a) *Pinus wallichiana* , (b) *Indigofera trita* , and (c) *Leontice eversmamnii* relation to different environmental variables, along the Indus River, northern Pakistan.

This community was comprised of seven sampling sites covering an altitude range of 2010–3110 m. The ISA highlighted *Pinus wallichiana* , *Indigofera trita* , and *Leontice eversmamnii* as the topmost indicator species of this plant community (Figure 4). These species were under the influence of higher concentrations of potassium (0.1–13.1 mg g^−1^), ORP (63.8–115.5), sand (43%–68%), and silt (20.4%–46.2%) (Table 1). The dominant tree species in this community were *P. wallichiana* (167.8 plant ha^−1^) and *C. deodara* (51.3 plant ha^−1^), while the dominant herbs were *Rosa webbiana* (44.2 plant ha^−1^) and *Phyllanthus parvifolius* (28.9 plant ha^−1^). The most abundant herbaceous species were *Poa polycolea* (49.5 plant ha^−1^) and *Aquilegia nivalis* (43.4 plant ha^−1^). The rare species included *M. pulegium* (1.7 plant ha^−1^), *Leontice eversmamnii* (1.5 plant ha^−1^), *Asparagus filicinus* (1.3 plant ha^−1^), *T. wallichiana* (0.7 plant ha^−1^), *Conyza bonarienss* (0.4 plant ha^−1^). The soil pH of this plant community varies from 6.7 to 7.4, salinity (0%–0.1%), MWHC (7.3%–16.1%), soil moisture (14.6%–32.1%), carbon content (0.7%–2.4%), clay (2%–24.4%), calcium (1–13.9 mg g^−1^), sodium (0.3–27.7 mg g^−1^), nitrogen (2.6%–2.9%), and phosphorus (4–10 mg g^−1^).

#### 3.1.3 PPV Community

**FIGURE 5:**
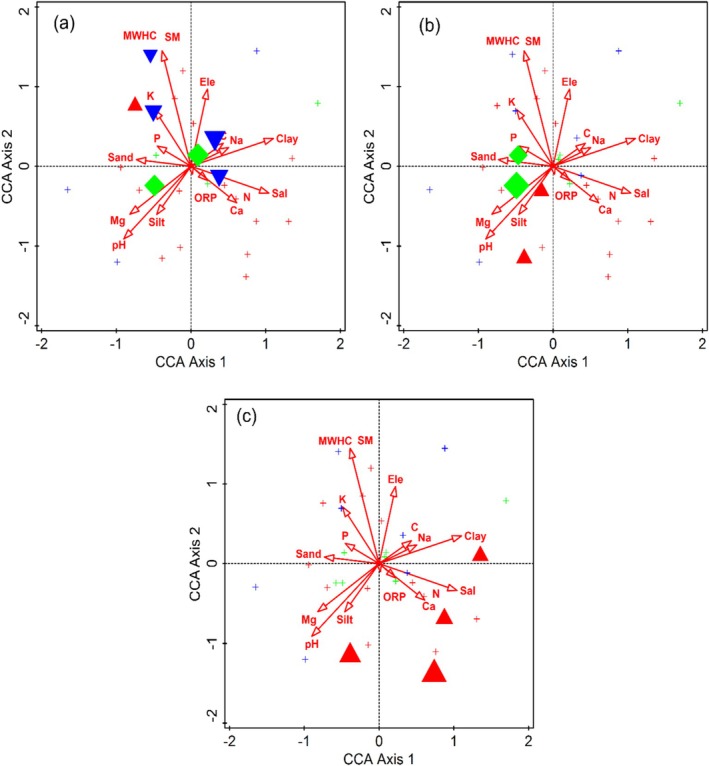
Attribute plots of indicator species (a) *Pinus gerardiana* , (b) *Phyllanthus parvifolius*, and (c) *Valeriana jatamansi* in relation to various environmental variables, along the Indus River, northern Pakistan.

This community was confined to seven different sampling sites at high altitudes ranging from 2390 to 3380. The Key indicator species of this community were *Pinus gerardiana* , *Phyllanthus parvifolius*, and *Valeriana jatamansi* (Figure 5). These species were indicators of higher concentration of sodium (2.2–30.5 mg g^−1^), MWHC (7.9%–19.6%), soil moisture (15.8%–39.2%), and carbon content (0.4%–2.9%) (Table 1). The dominant tree species of this community were *C. deodara* (126.8 plant ha^−1^), and *Pinus gerardiana* (49.5 plant ha^−1^). The dominant shrub species were *Indigofera trita* (9.9 plant ha^−1^), and *Astragalus himalayannus* (7.7 plant ha^−1^). Among herbaceous species, *Cleome scapose* (636.5 plant^−1^) and *C. bonarienss* (144 plant^−1^) were the most abundant. Rare species in this community included *M. longifolia* (1.7 plant ha^−1^), *Pteridium aquilinum* (1.7 plant ha^−1^), *Ranunculus laetus* (1.4 plant ha^−1^), *Rheum spiciforme* (1.4 plant ha^−1^), *Podophyllum emodi* (1.3 plant ha^−1^), *Leontice eversmamnii* (1.1 plant ha^−1^), *Picea smithiana* (0.4 plant ha^−1^). The soil condition of this plant community ranges from 6.7–7.4 pH, salinity (0.01%–0.1%), ORP (52.8–93.1), sand (39.2%–58.6%), silt (27.6%–45.6%), clay (0.8%–22.6%), calcium (2.2–24.4 mg g^−1^), magnesium (0.1–5.2 mg g^−1^), potassium (0.1–5.1 mg g^−1^), and nitrogen (2.7%–3.3%).

### 3.2 Diversity Pattern Along the Altitudinal Gradients

**FIGURE 6:**
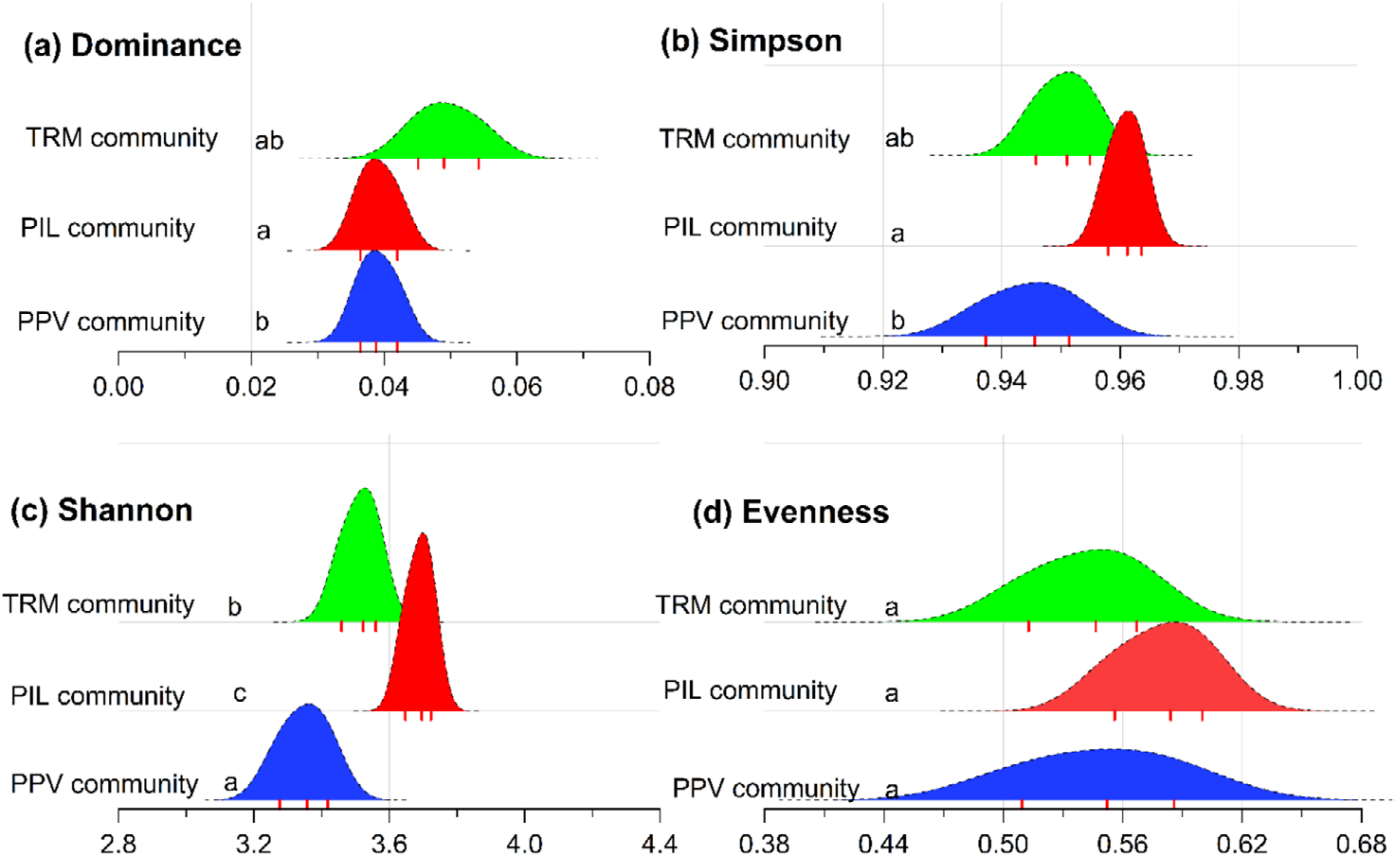
Ridgeline plots for diversity variation among the plant communities (TRM, PIL, and PPV) along the Indus River, northern Pakistan. The lower letters on the left side of each figure represent the significant difference calculated with Tukey post hoc test.

The Tukey post hoc test for Dominance index demonstrates significant variation among the plant communities (Figure 6). The TRM community reveals the highest mean dominance (0.05 ± 0.01) than other communities. The PIL community exhibits the highest mean Simpson (0.96 ± 0.1), Shannon (3.69 ± 0.03), and Evenness indices (0.58 ± 0.02). Among the extracted communities, the PPV community obtained the lowest Dominance, Simpson, Shannon, and Evenness indices (Figure 6).

#### 3.2.1 Environmental Gradient Analysis Through Canonical Correspondence Analysis and Detrended Correspondence Analysis (DCA)

**FIGURE 7:**
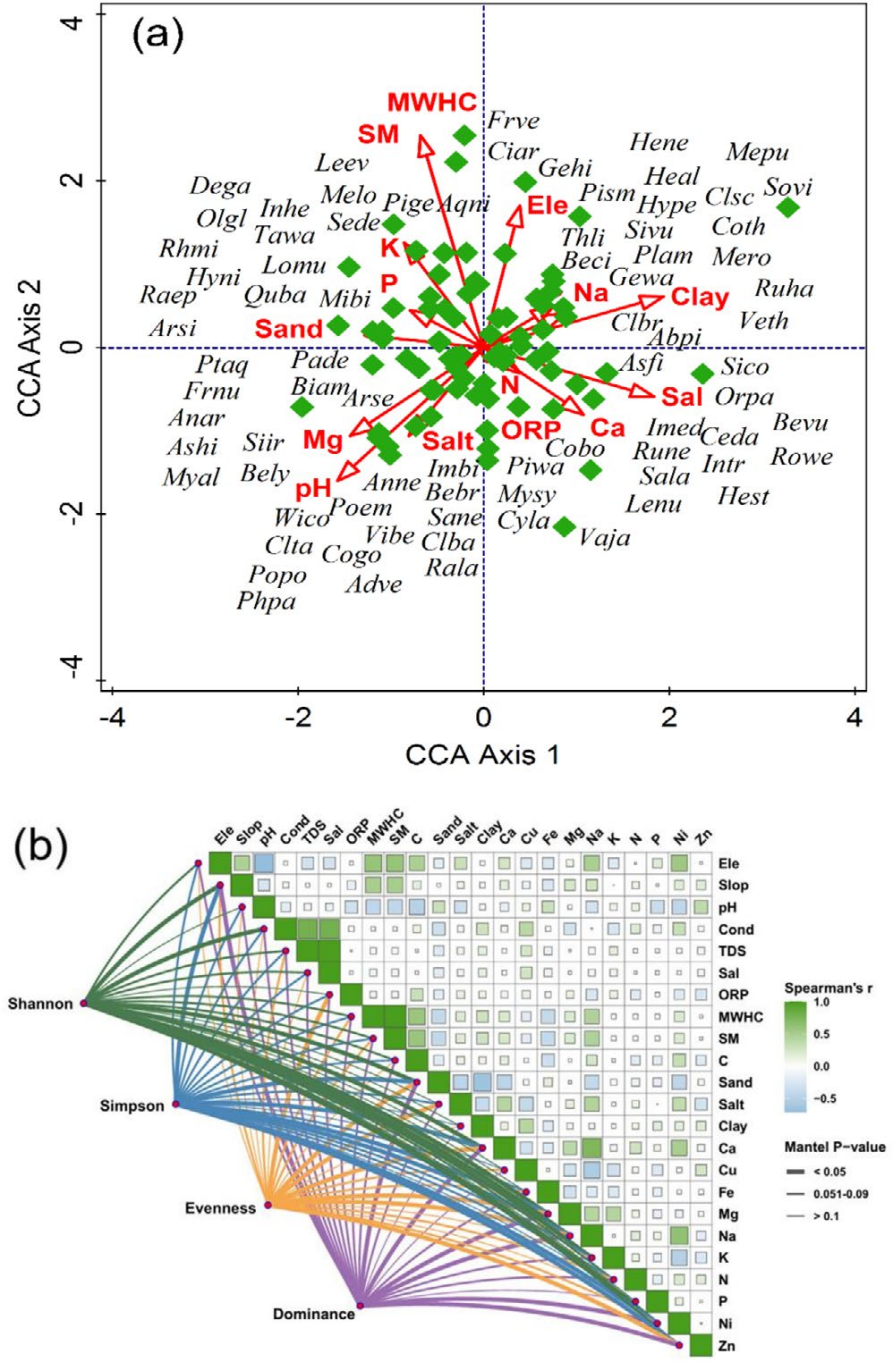
(a) Canonical correspondence analysis (CCA) of vegetation in relation to various environmental variables (b) correlation matrix among environmental variables across the Indus River, northern Pakistan.

**FIGURE 8:**
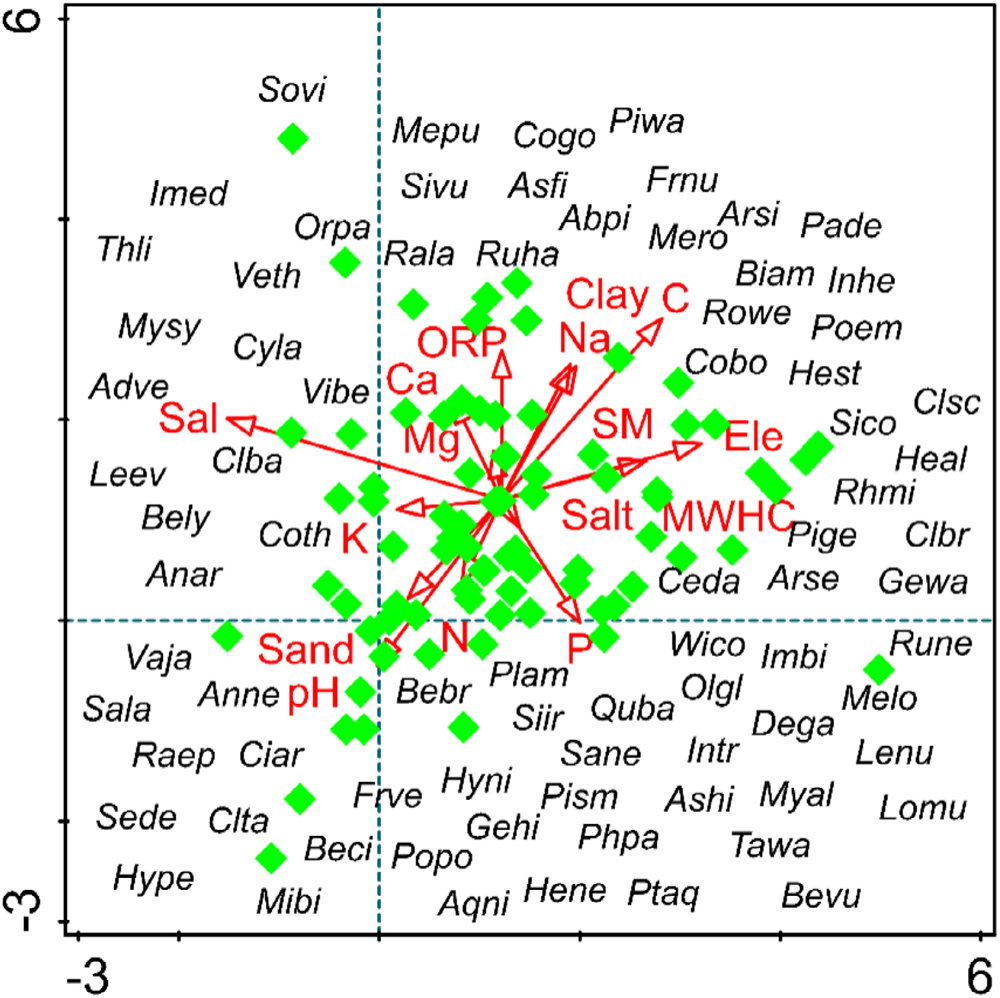
Detrended correspondence analysis (DCA) plot shows the distribution of 78 species in relation to various environmental variables.

The influence of environmental variables on plant species association and distribution pattern was determined through CCA and DCA (Figures 7 and 8). The first and second axes of the CCA ordination explained 17.89% of the cumulative variance. These axes accounted for 36.27% of the fitted cumulative variance. The CCA biplot of the first quadrant (top‐left quadrant) indicates that most species were assembled around maximum water holding capacity (MWHC), moisture content (MC), sand, phosphorus (P), potassium (K), and electrical conductivity (EC). In the second quadrant (top‐right quadrant), most plant species were associated with altitude, sodium (Na), and clay. In the third quadrant (lower‐left quadrant), pH, magnesium (Mg), and silt showed a substantial influence on plant species associations. In the fourth quadrant (lower‐right quadrant), plant species were clustered around oxygen‐reduction potential (ORP), calcium and salinity. Mantel test was performed to assess the variation in environmental variables along the altitudinal gradient and their influence on species diversity. The network heatmap, which reflects the results of Mantal test reveals that some of the environmental variables like altitude, pH, Conductivity, MWHC, SM, Salt, Ca, Mg, and Na have substantial influence on species diversity (Figure 7b). This indicates that variation are found in species diversity along the altitudinal gradient of Indus River.

Detrended Correspondence Analysis (DCA) was conducted to explore the distribution patterns of 78 plant species across the studied sampling sites (Figure 8). The DCA biplot reveals environmental gradients (red arrows) and species distribution (green diamonds). The DCA axes explain a total of 23.892% of the variance, with eigenvalues ranging from 0.3562 to 0.1498 and a total inertia of 4.22. The first DCA axis exhibited the maximum gradient length (3.07) with an eigenvalue of 0.3562 (Table 2). The second, third, and fourth axes revealed gradient lengths of 3.12, 2.68, and 2.33 with corresponding eigenvalues of 0.275, 0.2282, and 0.1498 respectively. The DCA ordination illustrated that various environmental variables, such as carbon, salinity, pH, and soil texture, substantially influenced species distribution and association, as reflected by the arrows in the biplot.

**TABLE 2:** Summary table of Detrended Correspondence Analysis (DCA).

Axes	1	2	3	4	Total inertia
Eigenvalues	0.3562	0.275	0.2282	0.1498	23.892
Explained variation (cumulative)	8.43	14.94	20.34	23.89	
Gradient length	3.07	3.12	2.68	2.33	

## 4 Discussion

The investigation of floristic diversity and indicator species analysis (ISA) along altitudinal gradients across the Indus River basin provides important insights into plant community dynamics, species distribution patterns and the role of environmental factors in shaping high‐altitude ecosystems. In this study, a total of 78 plant species belonging to 40 families were recorded. The widespread family was Asteraceae (14.1%), followed by Pinaceae (9.1%), Lamiaceae (8.1%), and Berberidaceae (6.4%). These families have large ecological amplitudes, as recorded from various mountainous regions of Pakistan (Sultan‐Ud‐Din et al. 2016; Irfan et al. 2018; Hussain et al. 2019; Anwar et al. 2023). Asteraceae, Lamiaceae, Rosaceae, and Poaceae are predominantly notable for their adaptability for diverse microclimates (Öztürk et al. 2022; Rahman et al. 2022). Most of the noted plant species were herbaceous (75.6%), followed by shrubs (15.4%) and trees (8.9%), indicating ecological complexity of the bank of Indus River, and the influence of altitudinal gradients and soil factors on species distribution patterns. The results of our study were consistent with those reported from other mountainous adjacent regions (Hussain et al. 2019; Anwar et al. 2023; Dani et al. 2023; He et al. 2023), emphasizing the significance of topographic and edaphic factors on species diversity and association.

The distribution of life and growth forms provides important information about plant adaptive strategies along the altitudinal gradients (Manan et al. 2022). Chamaephytes were the dominant life form that reflect the subalpine and alpine stress tolerance strategy followed by phanerophytes. The dominance of chamaephytes showed adaptation to harsh climatic mountainous conditions (Maroua 2022; Zeb et al. 2026). Similar chamaephytes dominance has been reported from the other Himalayan and temperate mountainous ecosystems where climatic severity increases with elevation (Abbas et al. 2017; Khan et al. 2018). The relatively high proportion of geophytes indicated adaptation to seasonal climatic fluctuations (Howard et al. 2019). Whereas, phanerophytes usually represented coniferous tree species ( *P. wallichiana* , *C. deodara* and *P. gerardiana* ) were more prominent at lower and mid elevations with environmental conditions comparatively moderate in the studied region. In the current study, herbaceous species dominated the flora followed by shrubs and tree species. The predominance of the herbaceous layer indicated short life cycles and flexible reproductive strategies along altitudinal and distinct soil conditions (Laiolo and Obeso 2017; Kermavnar et al. 2022). Most recorded herbaceous vegetation in the current study was perennial with rapid regeneration during favorable periods and significant persistence under harsh climatic conditions and anthropogenic pressure.

### 4.1 Altitudinal Gradient Analysis

The altitudinal gradients have substantial influence on edaphic factors, resulting significant variations in plant species association and distribution pattern. In this study, we observed significant changes in plant diversity and distribution across the sampling sites. Plant communities often change along the altitudinal gradients due to change in environmental factors (Khan et al. 2017). The altitudinal gradients impose simultaneous changes in soil development, moisture contents and nutrient availability which limit species performance based on their trait requirement and tolerance (Ali, Khan, et al. 2023; Anwar et al. 2023). In such systems, plant species with broad ecological niches may persist across a wide range of environmental conditions while other becomes restricted to specific elevational range (Chen et al. 2024). Hence, the observed differentiation in plant communities' assemblage along the gradients reflects a combination of niche based environmental filtering and trait mediated responses to changing abiotic conditions. Similar patterns of diversity and composition along the altitudinal gradients have been documented in various geographical regions (Anwar et al. 2023; Dani et al. 2023; Gillani et al. 2024). The species‐area curves in our study further highlight the turnover in plant diversity and composition particularly from the sampling site 3 to sampling site 29. This analysis emphasized that altitudinal gradient limits the plant communities and their associated Indicator species of Indus River. The cluster analysis classified the sampling sites into three distinct plant communities along the altitudinal gradient. This highlights the significance of indicator species via supporting biodiversity and maintaining the ecosystem structure. In addition, these dominant species, along with other conifer species such *A. pindrow* and *P. smithiana* form the forest canopy, which regulate the microclimate conditions, supporting ground vegetation and maintaining the ecosystem (Aponte et al. 2013; Richter et al. 2022).

We also determined ISA for each community based on edaphic factors. While robust classification and ordination techniques have been applied on natural vegetation of the region (Khan et al. 2020), the application of ISA remains limited. This study provides a framework for vegetation ecologists to improve the accuracy and validation of their research. Our finding is aligned with the classification and indicator species analysis of vegetation conducted in another region of Pakistan (Khan, Khan, et al. 2016; Khan et al. 2017; Anwar et al. 2023). At lower altitudes (±2370 m), the first community was characterized by species such as *T. wallichiana, R. nepalensis*, *M. pulegium, B. brandisiana*, *B. lyceum, C*. *bonarienss*, *T. linearis* , *G. wallichianum, H. perforatum, V. thapsus, C. govaniana*, and *H. altaicus* . The recorded species in our study were consistent with the results of other studies conducted in various regions of Pakistan (Sultan‐Ud‐Din et al. 2016; Bukhari et al. 2021; Anwar et al. 2023). The second community, located at moderate altitude (±2582 m), was characterized by *P. wallichiana* , *I. trita* , *L*. eversmamnii, *R. webbiana* , *P. parvifolius* , *P. polycolea*, *A. nivalis* , *M. pulegium* , and *A. filicinus*. At higher altitude (±2812 m), the third community was defined by unique species such as *P. gerardiana* , *P. parvifolius* , *V. jatamansi*, *I. trita* , *A. himalayannus, C. scapose, M. longifolia*, *P. aquilinum* , *R. laetus, R. spiciforme*, and *P. emodi* . It was observed that species diversity was highest at mid‐altitudes (±2582 m), whereas lower diversity was recorded at higher elevations. This pattern indicated variation in measured edaphic variables along the altitudinal gradient. The CCA ordination and Mantel test results showed that the soil moisture, potassium, sodium, pH, carbon content, and soil texture significantly influenced species diversity and distribution pattern. The diversity peak at mid‐elevations may therefore reflect favorable combinations of these measured soil properties (Dani et al. 2023). In contrast, shifts in soil moisture and nutrient availability at higher elevations may restrict species composition or establishment and reduce overall diversity. These results are in close harmony with the findings of (Khan, Khan, et al. 2016; Dani et al. 2023).

### 4.2 Role of Edaphic Variables

The ecological indicator species recognized through CCA ordination biplots showed a marked relationship with topographic and edaphic factors. These indicator species vary across the altitudinal gradient. Like topographic factors (altitude and slope), the edaphic factors also play a considerable role in shaping species composition and determining indicator species for each community. Community at lower elevation was associated with soils rich in calcium, pH and salinity, whereas at moderate elevation it showed correlation with potassium, ORP, sand, and silt. It reflects increased weathering processes and redistribution of exchangeable cations along the slope. Studies reported that the altitude impacts the soil pH and texture and hence had a significant influence on species composition and distribution pattern (Bui 2013; Wu et al. 2020; Hamid et al. 2021). Sodium, moisture and carbon contents were found to be increased at high altitudes and correlate with distinct species association or community formation. The mountainous soil usually experiences greater organic matter accumulation and altered cation mobility due to the lower decomposition rates and increased precipitation (Zhongsheng et al. 2023). Such processes can impact the base cation leaching, nutrient redistribution and soil structure development (Ewunetu et al. 2025). Therefore, the observed Ca, K and Na gradients reflect the altitudinal structured pedogenic process, including differential leaching, weathering and organic matter dynamics which in turn regulate the plant species assemblage through edaphic variables (Mumshad et al. 2021). Similar results were also recorded in the studies conducted at the Western Himalayan region (Kewlani et al. 2021; Gillani et al. 2024). These variations in environmental variables have a substantial contribution to shaping species associations and diversity patterns. Some environmental variables such as clay content and certain trace elements showed relatively weak correlations with species distribution in the ordination analysis. Since these variables did not emerge as significant drivers in the CCA or Mantel tests, they likely play a secondary role compared to key factors such as soil moisture, salinity, pH and major cations. Therefore, the primary ecological patterns observed in this study were best explained by the dominant edaphic gradients identified through multivariate analysis (Haq et al. 2022; Ali, Muhammad, et al. 2023). This study enhances our understanding of life form and ISA of the forest's vegetation along the Indus River, northern Pakistan, indicating that species associations and distributions are primarily controlled by altitudinal gradients in general, and edaphic factors in particular. The analysis of ISA also provides valuable insight into the underlying mechanism of ecological processes via shaping species patterns in this region. However, more in‐depth study is required to explore further ecological aspects and their significance on species association and distribution.

### 4.3 Limitation of the Study

This study provides valuable insights into plant community patterns along an altitudinal gradient. However, there are few limitations i.e., environmental variables such as temperature, precipitation, and anthropogenic pressure were not directly measured. These unmeasured drivers may interact with the edaphic gradients and influence species distributions. The indicator species analysis (ISA) and clustering results are dependent on the selection of cut‐off thresholds and data types (presence, absence vs. abundance), which may influence community description. The density estimates were based on quadrat sampling and although standardized may not fully capture spatial heterogeneity. Therefore, future research work may incorporate climatic data, experimental validation and trait‐based analyses to further explain the mechanisms driving plant community assembly in the studied region.

## 5 Conclusions

This study examined plant community structure and soil associations along an altitudinal gradient using multivariate classification and ordination approaches. The cluster analysis classified the sampling sites into three different plant communities each with different species as significant indicators under the applied indicator species analysis criteria. Altitudinal gradients, soil pH, salinity, oxygen reduction potential, carbon content, soil texture, calcium, potassium and sodium were the primary environmental drivers shaping these species communities/associations and their associated indicator species. *T. wallichiana, R. nepalensis* and *M. pulegium* were key indicators of higher concentration of calcium, pH and salinity. *P. wallichiana* , *I. trita* , and *L. eversmamnii* indicated higher concentration of potassium, ORP, sand and silt. *P. gerardiana* , *P. parvifolius* and *V. jatamansi* were indicative of higher concentration of sodium, MWHC, soil moisture and carbon contents. These indicator species represent statistically derived associations/communities with specific soil chemical conditions rather than fixed ecological boundaries. Species diversity exhibited a mid‐altitudinal peak, while soil properties such as calcium, potassium and sodium showed structured variation along the gradient. These patterns concluded that the environmental factors related to edaphic contributes to community differentiation. The identified soil–plant associations may serve as preliminary reference points for ecological monitoring and hypothesis development. For instance, species associated with higher sodium or calcium levels could be used to guide targeted soil assessments in comparable habitats. However, their practical application for conservation or land management requires further validation through expanded sampling and experimental studies. Community description and indicator species identification were influenced by analytical choices, including clustering thresholds and data structure. The moderate explanatory power of the ordination analysis indicates that additional environmental drivers may influence species composition. Future research integrating climatic measurements, anthropogenic assessment and trait‐based approaches would strengthen understanding of vegetation dynamics along altitudinal gradients. This study provides statistically supported evidence of structured plant and soil relationships along an altitudinal gradient while highlighting the need for further mechanistic studies.

## Author Contributions

**Adam Khan:** conceptualization (equal), data curation (equal), formal analysis (equal), methodology (equal), visualization (equal), writing – original draft (equal), writing – review and editing (equal). **Sidra Saleem:** conceptualization (equal), writing – review and editing (equal). **Sahar Zaidi:** methodology (equal), writing – original draft (equal), writing – review and editing (equal). **Zeeshan Ahmad:** methodology (equal), writing – original draft (equal), writing – review and editing (equal). **Hamada E. Ali:** data curation (equal), formal analysis (equal), writing – original draft (equal), writing – review and editing (equal).

## Conflicts of Interest

The authors declare no conflicts of interest.

## Supporting information


**Data S1:** ece373228‐sup‐0001‐Supinfo.docx.

## Data Availability

Datasets supporting the findings of this study are available within the article and its Supporting Information.
