# Supplementary material for: Floristic Diversity and Indicator Species Analysis Along Altitudinal Gradients of the Upper Indus Basin, Northern Pakistan
Source: Ecol Evol. 2026 Mar 8;16(3):e73228. doi: 10.1002/ece3.73228 (PMC12968055; doi:10.1002/ece3.73228)
Supplement: Supplementary file 1 — Data S1: ece373228‐sup‐0001‐Supinfo.docx. [file ECE3-16-e73228-s001.docx]

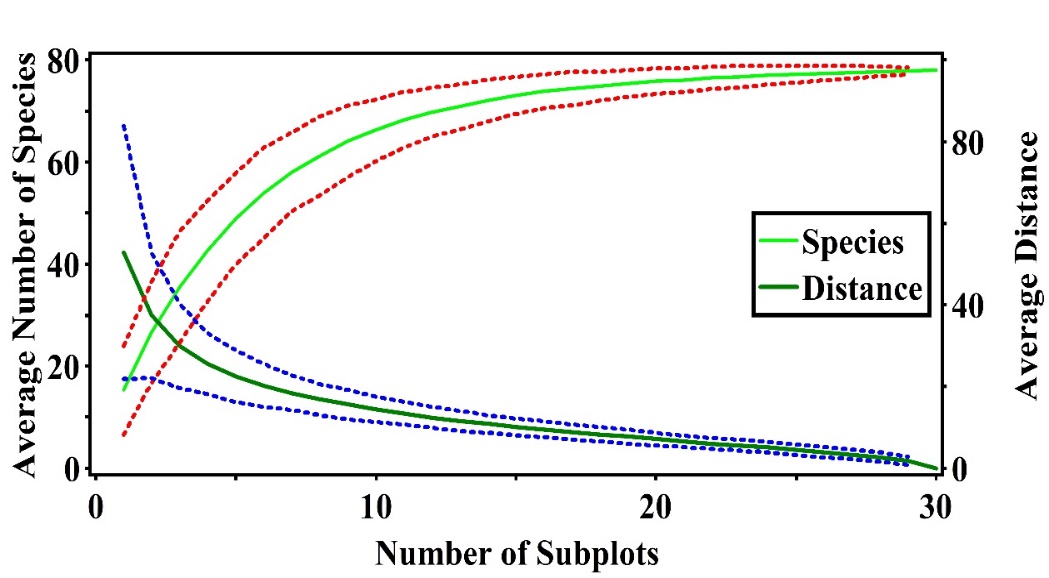


Supplementary Fig 1. Species-area curves (SAC) depict the appearance of novel species across sampled quadrats in the study area.


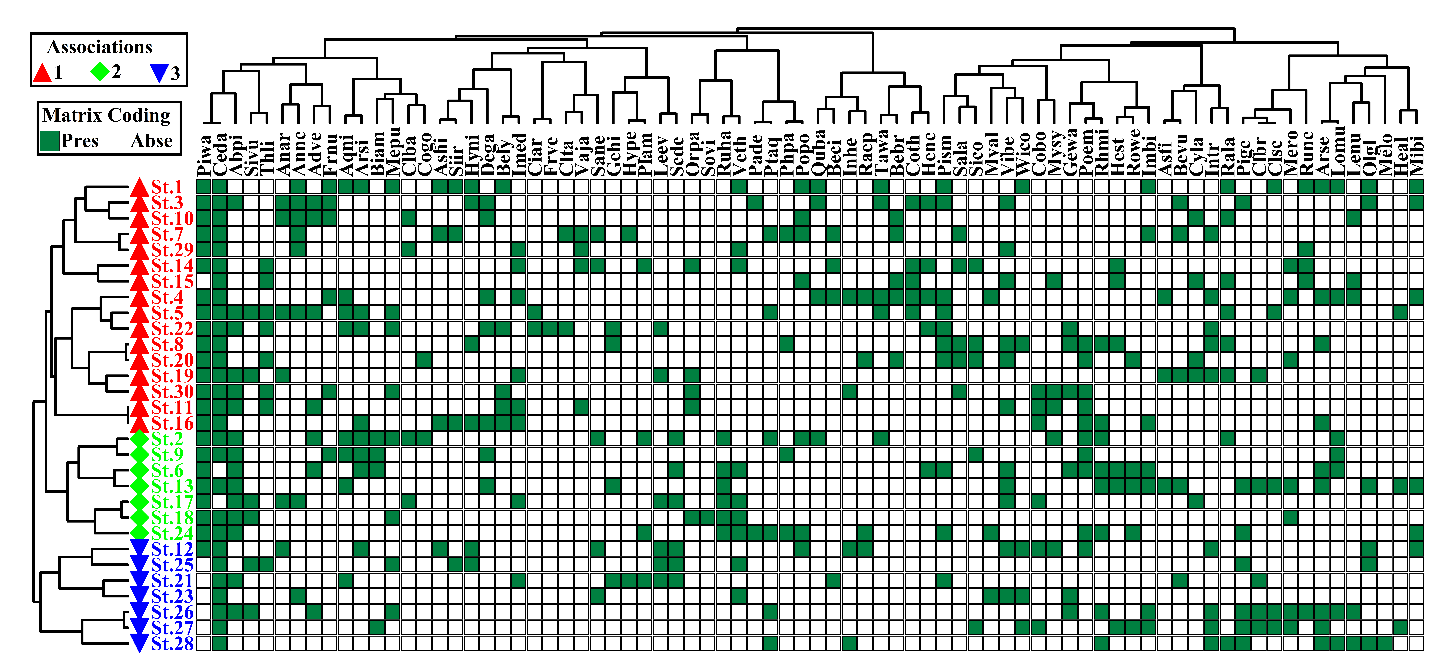


Supplementary Fig 2. Two-way cluster analysis (TCA) showing the distribution of plant species. The green dots demonstrate the presence of species, whereas the white dots reveal the absence of species in the sampling sites.

Supplementary Table 1 Species codes, family, life cycle, growth form, and lifeform recorded from the altitudinal gradient along the Indus River.

| S.N | Species | **Voucher number** | **Code** | **Family** | **Life cycle** | **Growth form** | **Life form** |
| --- | --- | --- | --- | --- | --- | --- | --- |
| 1 | *Pinus wallichiana* | GC.Herb.Bot.4387 | Piwa | Pinaceae | Perennial | Tree | Phanerophyte |
| 2 | *Cedrus deodara* | GC.Herb.Bot.4388 | Cede | Pinaceae | Perennial | Tree | Phanerophyte |
| 3 | *Quercus baloot* | GC.Herb.Bot.4389 | Quba | Fagaceae | Perennial | Tree | Phanerophyte |
| 4 | *Picea smithiana* | GC.Herb.Bot.4390 | Pism | Pinaceae | Perennial | Tree | Phanerophyte |
| 5 | *Abies pindrow* | GC.Herb.Bot.4391 | Abpi | Pinaceae | Perennial | Tree | Phanerophyte |
| 6 | *Pinus gerardiana* | GC.Herb.Bot.4392 | Pige | Pinaceae | Perennial | Tree | Phanerophyte |
| 7 | *Taxus wallichiana* | GC.Herb.Bot.4393 | Tawa | Pinaceae | Perennial | Tree | Phanerophyte |
| 8 | *Angelica archangelica* | GC.Herb.Bot.4394 | Anar | [Apiaceae](http://en.wikipedia.org/wiki/Apiaceae) | Biennial | Herbs | Cryptophyte |
| 9 | *Adiantum venustum* | GC.Herb.Bot.4395 | Adve | Pteridaceae | Annual | Herbs | Cryptophyte |
| 10 | *Anaphalis nepalensis* | GC.Herb.Bot.4396 | Anne | Asteraceae | Perennial | Herbs | Cryptophyte |
| 11 | *Aquilegia nivalis* | GC.Herb.Bot.4397 | Aqni | Ranunculaceae | Perennial | Herbs | Cryptophyte |
| 12 | [*Arenaria serpyllifolia*](http://palni.contentdm.oclc.org/cdm/search/collection/herbarium4/searchterm/Arenaria%20serpyllifolia%20L./mode/exact) | GC.Herb.Bot.4398 | Arse | [Caryophyllaceae](http://palni.contentdm.oclc.org/cdm/search/collection/herbarium4/searchterm/Caryophyllaceae/mode/exact) | Annual | Herbs | Therophyte |
| 13 | *Artemisia sieversiana* | GC.Herb.Bot.4399 | Arsi | Asteraceae | Biennial | Herbs | Chamaephyte |
| 14 | *Asparagus filicinus* | GC.Herb.Bot.4400 | Asfi | [Asparagaceae](https://en.wikipedia.org/wiki/Asparagaceae) | Perennial | Herbs | Cryptophyte |
| 15 | *Astragalus himalayannus* | GC.Herb.Bot.4401 | Ashi | Papilionaceae | Perennial | Shrub | Chamaephyte |
| 16 | *Berberis brandisiana* | GC.Herb.Bot.4402 | Bebr | Berberidaceae | Perennial | Shrub | Phanerophyte |
| 17 | *Berberis lycium* | GC.Herb.Bot.4403 | Bely | Berberidaceae | Perennial | Shrub | Phanerophyte |
| 18 | *Berberis vulgaris* | GC.Herb.Bot.4404 | Bevu | *Berberidaceae* | Perennial | Shrub | Phanerophyte |
| 19 | *Bergenia cilliata* | GC.Herb.Bot.4405 | Beci | Saxifragaceae | Perennial | Herbs | Geophyte |
| 20 | *Bistorta amplexicaulis* | GC.Herb.Bot.4406 | Biam | Polygonaceae | Perennial | Herbs | Geophyte |
| 21 | *Cirsium arvense* | GC.Herb.Bot.4407 | Ciar | [Asteraceae](https://en.wikipedia.org/wiki/Asteraceae) | Perennial | Herbs | Chamaephyte |
| 22 | *Clematis barbellata* | GC.Herb.Bot.4408 | Clba | Ranunculaceae | Perennial | Herbs | Phanerophyte |
| 23 | *Clematis tangutica* | GC.Herb.Bot.4409 | Clta | Ranunculaceae | Perennial | Herbs | Phanerophyte |
| 24 | *Cleome brachycarpa* | GC.Herb.Bot.4410 | Clbr | Capparaceae | Perennial | Herbs | Chamaephyte |
| 25 | *Cleome scaposa* | GC.Herb.Bot.4411 | Clsc | Capparaceae | Annual | Herbs | Therophyte |
| 26 | *Conyza bonarienss* | GC.Herb.Bot.4412 | Cobo | Asteraceae | Annual | Herbs | Therophyte |
| 27 | *Corydalis govaniana* | GC.Herb.Bot.4413 | Cogo | Fumariaceae | Perennial | Herbs | Chamaephyte |
| 28 | *Cousinia thomsonii* | GC.Herb.Bot.4414 | Coth | Asteraceae | Perennial | Herbs | Chamaephyte |
| 29 | *Cynoglossum lanceolatum* | GC.Herb.Bot.4415 | Cyla | Boraginaceae | Perennial | Herbs | Chamaephyte |
| 30 | *Desmodium gangeticum* | GC.Herb.Bot.4416 | Dega | Papilionaceae | Perennial | Shrub | Chamaephyte |
| 31 | *Fragaria nubicola* | GC.Herb.Bot.4417 | Frnu | [Rosaceae](https://en.wikipedia.org/wiki/Rosaceae) | Perennial | Herbs | Geophyte |
| 32 | *Fragaria vesca* | GC.Herb.Bot.4418 | Frve | *Rosaceae* | Perennial | Herbs | Geophyte |
| 33 | *Geranium himalayense* | GC.Herb.Bot.4419 | Gehi | Geraniaceae | Perennial | Herbs | Geophyte |
| 34 | *Geranium wallichianum* | GC.Herb.Bot.4420 | Gewa | *Geraniaceae* | Perennial | Herbs | Geophyte |
| 35 | *Hedera nepalensis* | GC.Herb.Bot.4421 | Hene | Araliaceae | Perennial | Herbs | Phanerophyte |
| 36 | *Heliotropium strigosum* | GC.Herb.Bot.4422 | Hest | Boraginaceae | Perennial | Herbs | Therophyte |
| 37 | *Heteropappus altaicus* | GC.Herb.Bot.4423 | Heal | Asteraceae | Annual | Herbs | Therophyte |
| 38 | *Hyosymus nigir* | GC.Herb.Bot.4424 | Hyni | [Solanaceae](https://en.wikipedia.org/wiki/Solanaceae) | Annual | Herbs | Therophyte |
| 39 | *Hypericum perforatum* | GC.Herb.Bot.4425 | Hype | Hypericaceae | Perennial | Herbs | Chamaephyte |
| 40 | *Impatiens bicolor* | GC.Herb.Bot.4426 | Imbi | [Balsaminaceae](http://ceb.wikipedia.org/wiki/Balsaminaceae) | Annual | Herbs | Therophyte |
| 41 | *Impatiens edgeworthii* | GC.Herb.Bot.4427 | Imed | Balsaminaceae | Annual | Herbs | Therophyte |
| 42 | *Indigofera heterantha* | GC.Herb.Bot.4428 | Inhe | Papilionaceae | Perennial | Shrub | Chamaephyte |
| 43 | *Indigofera trita* | GC.Herb.Bot.4429 | Intr | Papilionaceae | Perennial | Shrub | Chamaephyte |
| 44 | *Leontice eversmamnii* | GC.Herb.Bot.4430 | Leev | [Berberidaceae](https://www.google.com.pk/search?biw=1366&bih=667&q=Berberidaceae&stick=H4sIAAAAAAAAAOPgE-LVT9c3NEwyzTUsKsiJV-LUz9U3MDOqNCvRssxOttJPyszPyU-v1M8vSk_MyyzOjU_OSSwuzkzLTE4syczPs8rITM9ILVJAFQUA1ML-plcAAAA&sa=X&ved=0ahUKEwjnu6-jqqnNAhVIWxQKHcHLAYcQmxMIgwEoATAV) | Perennial | Herbs | Geophyte |
| 45 | *Leucas nutans* | GC.Herb.Bot.4431 | Lenu | Lamiaceae | Annual | Herbs | Therophyte |
| 46 | *Lolium multiforum* | GC.Herb.Bot.4432 | Lomu | Poaceae | Ann, bi, peri | Herbs | Geophyte |
| 47 | *Mentha longifolia* | GC.Herb.Bot.4433 | Melo | [Lamiaceae](https://en.wikipedia.org/wiki/Lamiaceae) | Perennial | Herbs | Chamaephyte |
| 48 | *Mentha Pulegium* | GC.Herb.Bot.4434 | Mepu | Lamiaceae | Perennial | Herbs | Chamaephyte |
| 49 | *Mentha royleana* | GC.Herb.Bot.4435 | Mero | Lamiaceae | Perennial | Herbs | Chamaephyte |
| 50 | *Micromeria biflora* | GC.Herb.Bot.4436 | Mibi | Lamiaceae | Perennial | Herbs | Geophyte |
| 51 | *Myosotis alpestris* | GC.Herb.Bot.4437 | Myal | Boraginaceae. | Perennial | Herbs | Chamaephyte |
| 52 | *Myosotis sylvatica* | GC.Herb.Bot.4438 | Mysy | Boraginaceae | Perennial | Herbs | Chamaephyte |
| 53 | *Olea glandulifera* | GC.Herb.Bot.4439 | Olgl | Oleaceae | Perennial | Herbs | Phanerophyte |
| 54 | *Orthosiphon palliidus* | GC.Herb.Bot.4440 | Orpa | Lamiaceae | Perennial | Herbs | Chamaephyte |
| 55 | *Parasenecio delphiniifolius* | GC.Herb.Bot.4441 | Pade | [Asteraceae](https://en.wikipedia.org/wiki/Asteraceae) | Perennial | Shrub | Chamaephyte |
| 56 | *Phyllanthus parvifolius* | GC.Herb.Bot.4442 | Phpa | Euphorbiaceae | Perennial | Shrub | Chamaephyte |
| 57 | *Plantago amplexicaulis* | GC.Herb.Bot.4443 | Plam | [Plantaginaceae](http://en.wikipedia.org/wiki/Plantaginaceae) | Annual | Herbs | Therophyte |
| 58 | *Poa polycolea* | GC.Herb.Bot.4444 | Popo | Poaceae | Perennial | Herbs | Geophyte |
| 59 | *Podophyllum emodi* | GC.Herb.Bot.4445 | Poem | [Berberidaceae](http://en.wikipedia.org/wiki/Berberidaceae) | Perennial | Herbs | Cryptophyte |
| 60 | *Pteridium aquilinum* | GC.Herb.Bot.4446 | Ptaq | Dennstaedtiaceae | Perennial | Herbs | Geophyte |
| 61 | *Ranunculus laetus* | GC.Herb.Bot.4447 | Rala | Ranunculaceae | Perennial | Herbs | Geophyte |
| 62 | *Rheum spiciforme* | GC.Herb.Bot.4448 | Raep | *Polygonaceae* | Perennial | Herbs | Geophyte |
| 63 | *Rhynchosia minima* | GC.Herb.Bot.4449 | Rhmi | Febaceae | Perennial | Herbs | Chamaephyte |
| 64 | *Rosa webbiana* | GC.Herb.Bot.4450 | Rowe | Rosaceae | Perennial | Shrub | Phanerophyte |
| 65 | *Rumex hastatus* | GC.Herb.Bot.4451 | Ruha | Polygonaceae | Perennial | Shrub | Chamaephyte |
| 66 | *Rumex nepalensis* | GC.Herb.Bot.4452 | Rune | Polygonaceae | Perennial | Shrub | Chamaephyte |
| 67 | [*Satyrium nepalense*](http://en.wikipedia.org/wiki/D.Don) | GC.Herb.Bot.4453 | Sane | [Orchidaceae](http://en.wikipedia.org/wiki/Orchidaceae) | Perennial | Herbs | Geophyte |
| 68 | *Saussurea lappa* | GC.Herb.Bot.4454 | Sala | [Asteraceae](https://en.wikipedia.org/wiki/Asteraceae) | Perennial | Herbs | Chamaephyte |
| 69 | *Senecio deodrans* | GC.Herb.Bot.4455 | Sede | [Asteraceae](https://en.wikipedia.org/wiki/Asteraceae) | Annual | Herbs | Therophyte |
| 70 | *Sida cordifolia* | GC.Herb.Bot.4456 | Sico | Malvaceae | Perennial | Herbs | Chamaephyte |
| 71 | *Silene vulgaris* | GC.Herb.Bot.4457 | Sivu | [Caryophyllaceae](http://palni.contentdm.oclc.org/cdm/search/collection/herbarium4/searchterm/Caryophyllaceae/mode/exact) | Perennial | Herbs | Chamaephyte |
| 72 | [*Sisymbrium irio*](https://en.wikipedia.org/wiki/Carl_Linnaeus) | GC.Herb.Bot.4458 | Siir | [Brassicaceae](https://en.wikipedia.org/wiki/Brassicaceae) | Annual | Herbs | Therophyte |
| 73 | *Solidago virgaurea* | GC.Herb.Bot.4459 | Sovi | Asteraceae | Perennial | Herbs | Geophyte |
| 74 | *Thymus linearis* | GC.Herb.Bot.4460 | Thli | Lamiaceae | Perennial | Herbs | Cryptophyte |
| 75 | *Valeriana jatamansi* | GC.Herb.Bot.4461 | Vaja | Valerianaceae | Perennial | Herbs | Geophyte |
| 76 | *Verbascum Thapsus* | GC.Herb.Bot.4462 | Veth | Scrophulariaceae | Biennial | Herbs | Therophyte |
| 77 | *Viola betonicifolia* | GC.Herb.Bot.4463 | Vibe | [Violaceae](https://en.wikipedia.org/wiki/Violaceae) | Perennial | Herbs | Geophyte |
| 78 | [*Withania coagulan*](http://en.wikipedia.org/wiki/Dunal) | GC.Herb.Bot.4464 | Wico | [Solanaceae](http://en.wikipedia.org/wiki/Solanaceae) | Perennial | Herbs | Phanerophyte |
